# The Virtual Challenge: Virtual Reality Tools for Intervention in Children with Developmental Coordination Disorder

**DOI:** 10.3390/children8040270

**Published:** 2021-04-01

**Authors:** Federica Lino, Valentina Arcangeli, Daniela Pia Rosaria Chieffo

**Affiliations:** 1 Clinical Psychology Unit, Memory Clinic, IRRCS Fondazione Policlinico A. Gemelli, 00168 Roma, Italy; federica.lino.psy@gmail.com; 2 Clinical Psychology Unit, IRRCS Fondazione Policlinico A. Gemelli, 00168 Roma, Italy; valentina.arca@gmail.com; 3 Child Neuropsychiatry Unit, IRRCS Fondazione Policlinico A. Gemelli, 00168 Roma, Italy; 4 Faculty of Medicine and Surgery, Catholic University of Sacred Heart, 00168 Roma, Italy

**Keywords:** neurorehabilitation, cognitive enhancement in children, tech mediated rehabilitation, developmental coordination disorders, virtual reality

## Abstract

This narrative review highlights the latest achievements in the field of tele-rehabilitation: Virtual Reality (VR) and Augmented Reality (AR) serious games aimed at restoring and improving cognitive functions could be effectively used in Developmental Coordination Disorder Training. Studies investigating the effects of the abovementioned tech applications on cognitive improvement have been considered, following a comprehensive literature search in the scientific electronic databases: Pubmed, Scopus, Plos One, ScienceDirect. This review investigates the effects of VR and AR in improving space/motor skills through mental images manipulation training in children with developmental coordination disorders. The results revealed that in spite of the spreading of technology, actually only four studies investigated the effects of VR/AR tools on mental images manipulation. This study highlights new, promising VR and AR based therapeutic opportunities for digital natives now available, emphasizing the advantages of using motivational reward-oriented tools, in a playful therapeutic environment. However, more research in this filed is needed to identify the most effective virtual tool set for clinical use.

## 1. Introduction

In the last decades the number of tech applications for cognitive Rehabilitation in childhood has increased, thanks to technological innovation. Virtual Reality (VR) and Augmented Reality (AR) have arisen as a promising approach in the field of clinical rehabilitation. This narrative review aims at pointing out the most recent advances in the field of neuro-rehabilitation: It will focus on studies using VR and AR serious games in the treatment of Developmental Coordination Disorder (DCD) in childhood. Specifically, VR and AR training may be useful to enhance mental images manipulation such as rotation, spatial visualization, spatial orientation, and motor plan programming, offering children a constant feedback on their performance.

As a matter of fact, children with DCD show a reduced ability to imagine a motor act (esp. from a first-person perspective), which has been shown in research using mental limb rotation and visually guided pointing tasks [1]. Other works show that Mental Imagery (MI) ability is correlated with the ability to implement online corrections in healthy adults [2] and children with DCD [3].

Virtual environments have been found effective in representing further contexts for children to test their adaptive strategies and to improve responses. The present work has two advantages over previous works: First, it depicts the most recent state of the art of VR and AR application in children with DCD and, second, it identifies useful differences in terms of tools and methods applied to approach digital native’s neurorehabilitation. The main goal of this work is giving a view of the studies carried out so far and encouraging both researchers and clinicians to identify what should be further investigated to increase knowledge in this field of study.

## 2. Developmental Coordination Disorder

As described in the American Psychiatric Association’s (Washington, DC, USA) latest edition of the Diagnostic and Statistical Manual of Mental Disorders (DSM-5) [4], the child with DCD has motor coordination below expectations for his or her chronologic age and therefore may have been described as “clumsy” and may have had delays in early motor milestones, such as walking and crawling. Difficulties with coordination of either gross or fine motor movements, or both, could interfere with academic achievement or activities of daily living. Coordination difficulties do not relate to a medical condition or disease (e.g., cerebral palsy, muscular dystrophy, visual impairment, or intellectual disability). If intellectual disability is existing, the motor difficulties are in excess of those expected for the child’s IQ. In the previous DSM edition (DSM-IV-TR) [5] DCD was included under the broad category of “learning disorders”; In DSM-5, it is subcategorized as a motor disorder within the broader category of “neurodevelopmental disorders.” An additional criterion included in DSM-5 is that the onset of symptoms occurs during the developmental period.

All children with DCD show some impairment in motor learning and in new motor skill acquisition, as opposite to adult apraxia which is a disorder in the execution of already learned movements. There is no consensus on etiology of DCD. Intragroup approach through factor and cluster analysis highlighted that “motor impairment in DCD children varies both in severity and nature” [6]. Indeed, most studies have used screening measures of performance on some developmental milestones derived from global motor tests. Vaivre-Douret [6] has investigated in his review different functions together with standardized assessments, such as neuromuscular tone and soft signs, qualitative and quantitative measures related to gross and fine motor coordination and specific difficulties-academic, language, gnosic, visual motor/visual-perceptual, and attentional/executive in order to allow a better identification of DCD subtypes with diagnostic criteria and to provide an understanding of the mechanisms and of the cerebral involvement [7].

A systematic review of 16 studies involving school-aged children, published in 2011, showed significantly greater odds of developmental coordination disorder among children who had very low birth weights (<1500 g) or were very preterm (<32 week) than among age-matched controls born at term with normal birth weights (odds ratio [OR] 6.29, 95% confidence interval [CI] 4.37–9.05 to 8.66, 95% CI 3.40–22.07) [8]. Boys are 1.7 to 2.8 times more likely than girls to have the disorder [9].

In a prospective, population-based cohort study in southwestern England, probable developmental coordination disorder was identified in 346 children aged 7–9 [10]. Risk factors associated with the disorder were difficulties with attention (OR 1.94, 95% CI 1.17–3.24), social communication (OR 1.87, 95% CI 1.15–3.04), repetition of nonwords (e.g., unfamiliar spoken nonwords that the child is asked to repeat) (OR 1.83, 95% CI 1.26–2.66), spelling (OR 2.81, 95% CI 2.03–3.90), and writing and reading (OR 3.35, 95% CI 2.36–4.77) [10].

### 2.1. Conventional Treatment in DCD

A plausible hypothesis to explain the compromised motor ability of children with Developmental Coordination Disorder (DCD) suggests a substantial deficit in their ability to utilize internal models for motor control. A dysfunction in this mode of control is thought to compromise motor learning capabilities [1].

Children with DCD have problems generating and/or monitoring a mental (action) representation of intended actions, termed the “internal modeling deficit” (IMD) hypothesis [11].

Internal modeling deficits (IMDs) have been proposed as a neurological cause of DCD [1]. According to the IMD hypothesis, the sensory-motor integration in the internal model is dysfunctional in children with DCD, which reduces their ability to use predictive motor control [1,12]. According to Nobusako [13] “before slow, sensory-motor feedback becomes available, internal models provide stability to the motor system by predicting the outcome of movements. This allows rapid online correction”.

Two approaches target the Internal Modeling: Mental Imagery (MI) and Action Observation (AO). According to Adams [14], the above mentioned are two faces of the same coin. Several studies combining MI and AO seem promising [15,16], confirming that MI, in Adam’s words, is “a prime modality that may serve motor intervention for children’s motor problems”. According to Marshall [17], an integrated MI/AO program through digital technology would be more promising, taking into account that children with DCD have difficulties in programming strategies by themselves only, while no difficulty is proved in learning once they have observed the correct strategy.

Other studies [18] showed that mental rotation (MR) ability is implicated in the successful execution of a motor task. This finding leads to the necessity of programming strategies and tools pointing to work on mental imagery in the treatment of DCD.

### 2.2. Mental Imagery

A mental image is a product of cognitive activity which enables to represent reality through recall, manipulation, reproduction of objects and events without sensorial stimulation [19]. People can experience mental images in all sensorial modalities. Therefore, if we can perceive in an auditorily or olfactorily way, “we can also have auditory, olfactory, tactile mental imagery” [20]. Nevertheless, the most common sensory modality through which we experience MI is vision [21]. Motor MI refers to a reproduction of a motor sequence of movements without perceptually witnessing that movement.

MI is not a single ability but emerges from a crossroads of singles abilities. Working memory has an important role in MI. According to Kosslyn [22], in order to generate mental images, a recall of images from long term memory to working memory is necessary. The image is then put into the “visuo-spatial-sketch pad” [23]. Working memory plays a central role in mental rotation tasks.

The same neuronal mechanisms underpinning simulation (imagery) are involved in real execution of actions [24]. As an evidence in visual MI, which is the most studied, earliest visual motor cortex (areas 17 and 18) is involved [25]. Other studies [18] have shown that mental rotation and motor performance tasks may share a similar subprocess.

MI have been investigated to examine the cognitive aspects linked to action and movement control. The main advantage in using MI in rehabilitation trainings is the possibility to significantly increase the number of task repetitions since we use a mental recall of motor tasks. Wilson [26] pioneered studies investigating the direct impact of MI training on DCD providing interesting results. Later, Adams [14] demonstrated the theoretically principled protocol for MI training in DCD. By using Internal Modelling of movements, the child is facilitated in predicting the consequences of movements. This is possible because, during the training, he has acquired information on his internal feeling of the movement to make predictions on movement outcomes.

### 2.3. Technology in DCD Rehabilitation

In the last decades, many tech tools enabling motor skills treatment in Neurodevelopmental Disorders and in the field of DCD were developed. This has been possible because of a growing interest in tailored tools, able to meet patient specific needs. In such a framework, technological tools emerge as motivating and stimulating devices, adaptable to a single child’s needs.

Telemedicine is currently developing in Italy and the National Healthcare System (NHS) has not exploited all the possibilities it offers yet [27]. In the field of Pediatric Medicine, telemedicine has the advantage of providing care and training in a non-medical environment [28,29]. Moreover, telemedicine allows custom-made training procedures, making daily interventions possible when needed. Research has contributed to a better understanding of the process underpinning children compliance to treatment. A game-like training setting has proved to be one of the most effective features for children in terms of motivation to the treatment.

In the last 20 years, gaming industry flourished and there was a combination between electronic games and neurorehabilitation research. “Serious Games” were born from this combination. With the term Serious Games we are referring to games whose peculiarity is not mere entertainment, but the empowerment of cognitive and motor function [30]. Video games training for rehabilitative purpose has been widely validated both in motor rehabilitation [31] and in cognitive empowerment and rehabilitation in several disorders [32,33,34] ensuring a similar efficacy compared to conventional treatments.

Regarding VR Intervention, Wilson [35] distinguishes between off the shelf tools (such as Wii fit) and specifically designed tools for rehabilitation (as Tele Rehab). Several studies have shown the effectiveness of off the shelf tools, combined with the convenience in terms of costs and usability: Nintendo Wii fit [36,37], Sony’s Playstation Eye Toy [38], Xbox 360 Kinect, and Playstation 3 [39].

Other studies explored the advantages of a specific design for tools in rehabilitation such as the AR Serious Game “Athynos” [40], an AR tool specifically developed to target cognitive/motor functions in Dispraxia.

### 2.4. Virtual Reality and Augmented Reality in DCD Treatment

Among various technologies explored to work on DCD, one recent and largely unexplored technology is Serious Games combined with VR and AR.

VR is defined as a three dimensional immersive and interactive experience occurring in real time [41]. AR can be described as a real environment which is ‘augmented’ by means of virtual objects through the use of computer graphic technology. Compared to VR “in which the users while immersed cannot see the real world, AR allows the user to see the real world with virtual objects superimposed upon or composited with the real world” [42].

VR has been successfully employed in the neurorehabilitation of several disease in adulthood after brain injury or stroke [43,44,45,46] and in the case of neurodegenerative diseases [47,48]. In childhood, VR and AR have been found effective in several conditions such as Autism Spectrum Disorder [49,50], Attention Deficit Hyperactivity Disorder (ADHD) [51,52,53,54], or cerebral palsy [55,56,57,58].

AR has been found effective in empowering coordination skills in children. A study by Avila Pesantez [40] investigated the effects of an AR Training using a specifically designed tool called Athynos. Athynos was a prototype designed according to practice standards proposed by experts in the field of Dispraxia. The objectives inspiring designers were the improvement of hand-eye coordination skills, feedback, interactivity and problem solving.

Despite the good chances offered by these technologies, only few studies applied VR and AR to DCDs. In 1987, Mc Clurg [59] proved that tridimensional object manipulation would lead to visuospatial skills improvement. In the 90’s Mc Comas [60] investigated the generalization effects of the improvement in visuospatial abilities gained through VR, confirming the generalization of the effects outside of the VR environment. The abovementioned results need to be further investigated [61] especially with regard to the extension of the generalization effect to more complex tasks.

In his study, Wilson [35] highlighted that VR could have an impact on different dimensions considered by the International Classification of Functioning [62]: Level of impairment, activity performance and skills, Participation, environment, personal factors (such as motivation or interests). With regard to training oriented to the empowerment of motor programming skills and mental rotation skills, VR enables children to manipulate 3D objects having an immediate feedback on task success into a realistic context. This represents one of most important advantages of VR technology in ecological terms.

## 3. Materials and Methods

This narrative review was implemented on the basis of a research conducted on major scientific online databases (PubMed, Scopus, Plos One, ScienceDirect). We included randomized controlled trials, reviews, systematic reviews, metanalysis, book chapters, and official guidelines from scientific associations published until December 2020. Our literature search was based on the following search terms: “Developmental Coordination Disorder” or “Coordination Disorder in children” or “motor coordination skills” or “mental rotation skills” or “mental images” or “mental images manipulation” combined with “VR treatment” or “VR/AR rehabilitation” or “VR intervention” or “Virtual remediation” or “Virtual Reality” or “Augmented Reality” or “virtual Reality training”.

To be considered for this review studies had to meet the following criteria. First, the study aim had to examine the impact of a VR or AR training on DCD in children; Second, the study protocol had to implement mental images manipulation such as rotation, spatial visualization, spatial orientation, and motor plan programming. Single case studies were not included in this study because of the lack of generality of obtained effects.

## 4. Results

From the literature search, only four studies specifically investigated the potential of VR or AR technology in DCD rehabilitation exploring mental images manipulation improvement.

In 2011, Straker [63] investigated the impact of a new VR video game on motor coordination skills in children with DCD. The study sample consisted of 30 children between the age of 10–12 with poor motor coordination ability (<15th percentile). Children underwent 16 weeks training in two different conditions: ‘Non active computer games’ and ‘active computer games’. Basic assumption was that children spend nowadays many hours playing video games and this reduces time spent in motor activities (with a consequent impact on motor competency). The first outcome measure was motor coordination ability, assessed by kinematic and kinetic three-dimensional motion analysis laboratory measures and two advanced mechanical technology force plates; Secondly, physical activity and sedentariness assessed through accelerometry have been considered; Coordination in daily life was measured by parent report questionnaire; Self-efficacy, anxiety, and mood outcomes were evaluated by self-report scales. The researchers’ hypothesis was that a change in the nature of movement of children playing computer games would lead to a beneficial outcome on their motor and physical ability and sense of confidence. The results of the study did not give and exhaustive answer on the matter of research.

In 2013, Ashkenazi [38] investigated a low-cost VR intervention program for children with DCD. The technological off-the-shelf VR tool selected for this study was the Playstation R 2 Eye Toy system. It consists of a camera for 2D gesture recognition which enables virtual object manipulation on a screen. A visual and acoustic signal provided an immediate feedback for success in the 8 games/tasks proposed. The small sample for this study consisted of 9 children between 4–6 years old. Subjects underwent a weekly 1 h training for 12 weeks. Trainings targeted motor planning, balance control, eye-hand coordination, and multitasking. A baseline and follow-up recording were obtained. Despite some weaknesses, such as the small sample and the lack of a control group, statistically significant results in the primary outcome measures-namely, Movement Assessment Battery for Children-2 (M-ABC-2) and DCD Questionnaire (DCD-Q)-emerged. Even though motivation was not directly measured in this study, authors pointed out the children’s enthusiasm for the device, supported by children preference for game-like training activities. For this reason, future studies could explore this feature in depth measuring variables such as motivation and enjoyment.

In 2020, a randomized controlled study [64] investigated the impact of a VR Serious Game training on motor control on a sample of 40 children with DCD, aged between 7 and 10 years. Specifically, the training targeted predictive control, described by the authors as the internal modelling of action in terms of MI and motor planning. The training consisted of 16 30 min session of Xbox 360 Kinect games administrated over an 8 week period. The selected VR device was unfamiliar to all children. Tasks to empower internal model control consisted in object manipulation and object control. Results demonstrated long lasting improvements, verified in the follow up phase. The outcome measures were MI skills (assessed through mental rotation task), action planning, and rapid and online control skills.

AR potentialities have also been investigated in the already mentioned study by Avila Pesantez [40], who compared two therapy methods (manual puzzle and AR) in 40 children (50% male and 50% female of average age 7.3 years). At each session the time of activities execution and the quality of performance for each student were registered. The study found significant difference between the two methods. The AR Serious game Athynos showed statistically significant gains in performance, described as less time spent on activities execution and less time spent in training, compared to the manual puzzle therapy. Improvement in motor level and hand and eye coordination were deducted from a boxplot of performance. There was no reference for a specific motor eye coordination assessment. Anthynos exploited the possibilities given by technology: It was programmed to give to children a constant feedback on achievements and tasks success rate. Lastly, the study pointed out another benefit of a similar approach in rehabilitation: Engaging children in proposed activities (taking into account that they are digital natives) ensuring as a consequence a good treatment compliance.

## 5. Discussion

The purpose of the present narrative review was twofold. First, we aimed to provide an up-to-date overview of the findings from VR/AR studies using mental images manipulation in DCD treatment for children. Second, we wanted to recommend researchers and clinicians worthy points to be additionally investigated in future research. Today, despite of the increasing use of technology in the clinical field, only four studies have been found interested in the role of VR and AR technologies in improving mental images manipulation for DCD treatment in children. Notwithstanding the literature is at a very early stage, some preliminary qualitative data can be recollected from the reviewed studies.

Results emerging from the presented studies, despite limitations, reveal a good potential in DCD treatment. Three out of the four studies presented in this review reported significant results in the outcome measures [38,40,64].

Straker [63] grounded his study approach on changing children’ habits involved in playing computer games. To provide positive gains in motor control, the study assumed that a change from non-active to active video games playing could provide a gain in motor performance. Avila Pesantez [40] and colleagues, differently from the other studies, designed their own AR tool exploiting the motivating power of web graphic design and gratifying children with colorful avatars. Two out of the four studies [38,64] investigated the effectiveness of a training designed through an off-the-shelf tool (PlayStation R and Xbox) showing that low-cost tools can be exploited in clinical treatment of DCD in children. Because of the use of commercial virtual gaming tools, rehabilitation experience could be extended out of the hospital setting.

All the studies included in the present work diverged in many methodological aspects, such as sample size, study timeline, type of tools designed for intervention. These differences limited to some extent the chance to determine a direct link between a specific intervention and different outcomes. The limitation of the studies included in the present review are mostly the sample exiguity or methodological differences. However, the abovementioned limitations are very common in pioneering studies exploring the use of new technological tools for neurorehabilitation.

To overcome this problem, the scientific community has been invited [61] to standardize methodologies in research for intervention in DCD through the use of the Template for Intervention Description and replication checklist by Hoffman [65]. This way better study procedures reporting will be possible and, consequently, a study comparison and replication will be possible. Randomized, controlled clinical studies with a larger sample are needed to give more accurate answers on effectiveness. At the current state of knowledge, well-structured VR and AR training programs implementing mental images manipulations could offer gains in terms of motor performance and perceived effectiveness.

Telemedicine is still under development in Italy but, since good results have been achieved from initial research, a great effort is required for researchers in this field of study. The potential of the use of VR/AR technology for children stands in the possibility of giving professional, daily treatment, intrinsically eliciting motivation and enjoyment. As a second advantage to be considered, children could be involved in well-structured game-like trainings outside of the hospital context.

Unfortunately, it could not be assessed which intervention is, among the others, more effective for a specific age-range. This was due to the fact that the studies investigated the efficacy of VR ad AR applications in different populations aged between four and twelve years (a wide age span). Greater attention should be dedicated in research to define whether a window of sensitivity is available or has to be agreed for this kind of treatments, since different age ranges have been considered in clinical studies conducted so far. Additionally, some attention should be dedicated to the longitudinal effects of virtual training in terms of improvement duration and outcome measures considered through life span. As an example, social involvement in children who underwent a DCD training during adolescence could be investigated.

The present review had some limitations. First, the goal of this work was to provide an up-to-date narrative overview of recent findings on the use of VR and AR Serious games training in DCD. To do this, we limited our search to a small amount of studies investigating the role of mental images on DCD rehabilitation. In addition, we considered only papers in English and we not considered single case studies because of the lack of generalizability of the outcomes. Moreover, as previously discussed, some methodological aspects of the reviewed studies also limited our findings (small sample size and different age range considered for intervention). Lastly, as is common knowledge, retention of learning skills for a certain time is a critical issue of any treatment. Future research on VR/AR interventions on DCD should include follow-up visits to evaluate the maintenance or the evolution of the benefits achieved over time.

## 6. Conclusions

VR and AR serious games training for DCD treatment are motivating tools, as they offer immediate feedback in very realistic game environments. Because of their features, VR and AR are among the most interesting tools researchers should look at to deliver tailored intervention for child’s needs, as required by recent scientific guidelines [61].

The present review suggests that VR and AR interventions could have some beneficial effects in treating DCD. This work encourages future trials to consider mental images manipulation through VR/AR tool as a possible target for interventions on children with DCD.

More research is needed in this field: First, to explore further chances offered by this technology as a strategic asset in the field of DCD and, second, to identify procedures and targets that best fit the needs in this specific clinical area. This approach could lead to soon determine best practices for using VR and AR technology in this field of study.
